# Effects of Ego-Resiliency on Interpersonal Problems among Nursing Students: The Mediating Effects of Aggression

**DOI:** 10.3390/healthcare10122455

**Published:** 2022-12-05

**Authors:** Sona Lee, Hye Young Ahn, Hye Seon Choi

**Affiliations:** 1 College of Nursing, Eulji University, Uijeongbu 11759, Republic of Korea; 2 College of Nursing, Woosuk University, Wanju 55338, Republic of Korea

**Keywords:** nursing students, psychological resilience, aggression, interpersonal relations, mediating

## Abstract

(1) Background: Despite that nursing college students are more diverse than those in other majors, many nurses experience interpersonal problems and difficulties in the process of forming relationships and contacting various people. The purpose of this study is to understand the mediating effects of aggression on the process of ego-resilience in interpersonal problems in nursing college students. (2) Methods: The subjects of this study were 182 nursing college students attending university in D metropolitan city. Data were collected from 23 October to 9 November 2018. The measurements were carried out using the Ego-Resiliency Scale, the Aggression Questionnaire—Korean Version (AQ-K), and the short form of the KIIP Complex Scale (KIIP-SC). Data were analyzed using descriptive statistics, t-tests, and ANOVA. The methods of Baron and Kenny were used to verify the significance of the mediating effect. (3) Results: There were significant correlations among ego-resiliency, aggression, and interpersonal problems. Aggression had a partial mediating effect on the relationship between ego-resiliency and interpersonal problems, and aggression was explained to a level of 23%. (4) Conclusions: To lower interpersonal problems among nursing students, it is necessary to develop education and programs to improve ego-resiliency and to control aggression.

## 1. Introduction

The period in which students attend college is an intermediate stage in the transition from childhood to adulthood [1]. Adolescents are classified as those under the age of 18, human beings are defined as beings in the environment, and the socio-cultural environment is said to have a great influence on personality development [1]. The period during which students attend college represents the end of adolescence, the maturity of adolescent development tasks, and the beginning of adult development tasks. The stage of adult development is in the sixth psychosocial development stage, and the main development task is intimacy versus isolation [2]. At this time, individuals strive to form intimate relationships with people other than family members, form satisfactory interpersonal relationships, and lay the foundation for personal maturity. Failure to successfully form intimate interpersonal relationships increases isolation and affects later developmental stages [1,2].

Students entering college live in diverse student groups, actively experience interactions that create new relationships, and grow into mature adults. In particular, nursing college students go to hospitals, public health centers, and industries and therefore meet more diverse people than college students from other majors due to their clinical practice. Interpersonal relationships, formed based on these experiences, are an important competency in becoming a nurse, communicating with the people faced in the medical field, and identifying and solving health problems [3]. In addition, it helps to work in a continuous relationship with doctors, medical cooperation departments, and nursing patients, forming complex relationships [4]. As such, it is important for nursing college students to experience and maintain positive interpersonal relationships as they become nurses after graduation and perform nursing tasks with people of various classes.

However, despite that nursing college students are more diverse than those in other majors, many nurses experience interpersonal problems and difficulties in the process of forming relationships and contacting various people. Nurses were found to be impacted by interpersonal difficulties and conflicts in peer indifference, careless words and attitudes [5], and friction with their bosses [6]. In addition, it has been reported in previous studies that interpersonal problems are a direct cause of nurse turnover, and that various programs and education are needed in schools to adapt to this aspect of hospital life [7]. As such, it is important to identify and manage interpersonal problems among nursing students so that they can form and maintain positive interpersonal relationships as future nurses.

Interpersonal problems include difficulties forming and maintaining satisfactory interpersonal relationships or an inability to cope with negative interpersonal relationships [8]. Humans experience happiness through sustaining, growing, and maintaining harmonious relationships in many forms of interpersonal relationships, but experiencing interpersonal problems and conflict situations with others leads to difficulties in adaptation and psychological discomfort [9]. Likewise, nursing college students can improve their satisfaction with clinical practice, reduce stress in clinical practice, and promote adaptation to college life by improving their interpersonal skills [10,11,12]. On the other hand, if it is difficult for them to form positive interpersonal relationships due to interpersonal problems, they will later experience difficulties, conflicts, and a loss of self-confidence when they become a nurse [7]. Therefore, intervention is needed to identify and reduce interpersonal problems.

Ego-resiliency is a variable related to interpersonal problems. Ego-resiliency refers to someone’s ability to control conflicts by themselves without showing behavioral or emotional problems in stressful, adverse, and threatening environments [13]. It is also the ability to successfully cope with stress and anxiety in an unpredictable situation, and to flexibly adjust and recover according to a given situation [14].

According to previous studies, ego-resiliency helps to cope and adapt successfully to stressful situations, such as interpersonal problems in college life [15]. In particular, previous studies with nursing college students confirmed that nursing college students’ ego-resiliency and success in interpersonal relationships were positively correlated [3] and that ego-resiliency had a positive effect on solving difficulties and problems in interpersonal relationships [16]. Meanwhile, the higher the aggression, the more difficult it is to maintain interpersonal relations [17,18] and the more difficult it is to overcome social difficulties [19]. In some studies, college students’ aggression has a partial mediating effect and affects interpersonal problems [20].

Most previous studies have reported on ego-resiliency and interpersonal problems [15,16,21], ego-resiliency and aggression [17,18], aggression and interpersonal problems [19,20], and mediating effects of aggression [8,19,20]. However, this is not enough to understand the mediating effect of aggression between ego-resiliency and interpersonal relationship problems, so it was necessary to verify it through this study. For this reason, this study aims to analyze aggression as a parameter to grasp the effect of ego-resiliency on interpersonal problems in nursing college students.

Therefore, this study was conducted to investigate the mediating effect of aggression on the self-resilience of nursing college students in the context of interpersonal problems. The specific purpose is as follows: We identify the general characteristics, ego-resiliency, aggression, and interpersonal problems of nursing college students, whereby ego-resiliency, aggression, and interpersonal problems are identified according to the general characteristics. We identify the correlations among ego-resiliency, aggression, and interpersonal problems. Finally, we grasp the mediating effect of aggression on the effects of ego-resiliency and interpersonal problems. In addition, the results are intended to be used as basic data in the development of nursing interventions to prevent interpersonal problems in the future by identifying interpersonal problems in nursing college students early.

## 2. Materials and Methods

### 2.1. Research Design

This study was a descriptive correlation study intended to grasp the relationship between ego-resiliency and interpersonal problems among nursing students and to confirm the mediating effect of aggression.

### 2.2. Subjects

For the subjects of this study, the researcher conveniently selected two places in D city. The subjects were nursing college students attending school who voluntarily answered the questionnaire. The sample size was calculated using the G-Power 3.1.9.2 Program, a sample size calculation program. Considering the conditions of an effect size of 0.15, significance level of 0.05, power of 0.95, and predictor of 9 required for multiple regression analysis, a minimum of 166 subjects were required. The survey was conducted on a total of 189 people considering the potential dropout rate. In total, 7 subjects did not respond to the questionnaire, so the recovery rate was 96.3%, and 182 responses were used as the final analysis data.

### 2.3. Instruments

#### 2.3.1. Ego-Resiliency

The Ego-Resiliency Scale [22] by Block and Kremen, developed by Yoo and modified with supplemented tools [23], was used. A total of 14 questions were measured on a 5-point Likert scale based on the evidence that a 4-point scale tool or a 5-point scale reflects the response to neutrality and can provide high-reliability results [24]. The higher the score, the higher the ego-resiliency. Cronbach’s α in Block and Kremen’s study was 0.76 [22], and Cronbach’s α in Yoo’s study was 0.67 [23]. In this study, Cronbach’s α was 0.82.

#### 2.3.2. Aggression (Aggression Questionnaire—Korean Version (AQ-K))

The Aggression Questionnaire (AQ) [25], developed by Buss and Perry, was translated into a Korean version by Seo and Kwon [26], which was used in this study. In total, there are 27 questions composed of 4 sub-areas: physical aggression, verbal aggression, anger, and hostility. The higher the score on the 5-point Likert scale, the higher the aggression. In Buss and Perry’s study, Cronbach’s α was 0.89 [25], and in Seo and Kwon’s study, Cronbach’s α was 0.86 [26]. In this study, Cronbach’s α was 0.91.

#### 2.3.3. Interpersonal Problems (Short Form of the KIIP Complex Scale (KIIP-SC))

The short form of the KIIP Complex Scale (KIIP-SC), developed and shortened by Horowitz et al. [27] and reconstituted by Alden, Wiggins, and Pincus as the Korean version of Hong Kong’s Inventory of Interpersonal Problems (IIP) [28], was used [29]. The 40 questions comprise 8 sub-domains: control dominance, self-centeredness, apathy, social restraint, non-assertiveness, over-compliance, self-sacrifice, and over-involvement. The higher the score on the 5-point Likert scale, the greater the interpersonal relationship problems. Cronbach’s α in Hong et al.’s study was 0.89 [29], and Cronbach’s α in this study was 0.93.

### 2.4. Data Collection

Data were collected from 23 October to 9 November 2018. We visited a university in D metropolitan city and explained the research purpose, necessity, data collection method, and procedure to nursing college students attending school, and then we began the research by seeking cooperation. Of the total 189 subjects, 182 subjects were included, with 7 responses excluded due to insufficient data. The questionnaire was prepared and collected immediately after distribution, and the time required to complete the questionnaire was about 15 min.

### 2.5. Data Analysis

The data collected for this study were analyzed statistically using IBM SPSS Statistics 24.0. The general characteristics, ego-resiliency, aggression, and interpersonal problems among nursing college students—the study subjects—were analyzed using technical statistics. Ego-resiliency, aggression, and interpersonal problems according to the general characteristics were analyzed by *t*-test and ANOVA. The correlations among ego-resiliency, aggression, and interpersonal problems among nursing college students were analyzed using Pearson’s correlation coefficient. The mediating effect of aggression on the relationship between ego-resiliency and interpersonal problems among nursing students was verified through the procedures of Baron and Kenny. The steps of Baron and Kenny’s hierarchical regression are as follows [30]: First, the significance between independent variables and parameters is tested. Second, the significance between independent and dependent variables is tested. Third, the significance among the independent variable, the parameter, and the dependent variable is tested. Fourth, the β value between the independent variable and the dependent variable and the β value among the independent variable, the parameter, and the dependent variable are compared. In this comparison, if the absolute value of the β value among the independent variable, the parameter, and the dependent variable is smaller, there is a partial mediating effect.

### 2.6. Ethical Considerations

This study was approved by the E-University Institutional Review Board (EU18-93). To ensure confidentiality, each questionnaire was distributed, filled out, and immediately sealed. During the questionnaire response, it was explained that the questionnaire could be abandoned and that answers would not be used for any purpose other than research.

## 3. Results

### 3.1. General Characteristics of the Subjects

The general characteristics of the subjects in this study are as follows. The proportion of female students in the nursing college was 82.4%, those in the fourth grade accounted for 33.0%, the third grade accounted for 26.4%, the second grade accounted for 24.7%, and the first grade accounted for 15.9%. Most subjects had no religion (61.5%), and most had two siblings (66.5%). Most of the subjects’ fathers (53.3%) and mothers (51.1%) were educated to an undergraduate level (Table 1).

### 3.2. Descriptive Statistics Regarding Ego-Resiliency, Aggression, and Interpersonal Problems

The descriptive statistics regarding ego-resiliency, aggression, and interpersonal problems, which are the variables of this study, are as follows (Table 2). The score for ego-resiliency was 3.38 ± 0.53 out of 5 points. Physical aggression was the lowest-scoring among the aggression types, with a score of 1.69 ± 0.58 out of 5, and anger was the highest-scoring, with 2.27 ± 0.68. The lowest scores for interpersonal problems were for control dominance with 1.70 ± 0.62 points and vindictive with 1.70 ± 0.67 points out of 5, while overly nurturing was the highest-scoring, with 2.55 ± 0.73 points.

### 3.3. Ego-Resiliency, Aggression, and Interpersonal Problems According to the Respondents’ General Characteristics

The focus of this study was on ego-resiliency, aggression, and interpersonal problems among nursing college students. The number of respondents with two siblings was 121 (66.5%), and there was a significant difference in ego-resiliency according to the number of siblings (F = 3.937, *p* = 0.021). As a result of the post-test, it was found that ego-resiliency was higher in the case of two siblings than in the case of one. In addition, there was a significant difference in interpersonal problems according to the grade level (F = 8.308, *p* < 0.001). As a result of the post-test, it was found that first graders had greater interpersonal problems than third and fourth graders, and second graders had greater interpersonal problems than fourth graders. There were 112 people (61.5%) who thought their economic status was middle, and there were significant differences in interpersonal problems according to economic status (F = 3.363, *p* = 0.037). As a result of the post-test, it was found that their interpersonal problem scores were higher than those of respondents who thought that their economic status was low (Table 3).

### 3.4. Correlations among Ego-Resiliency, Aggression, and Interpersonal Problems

The relationships among ego-resiliency, aggression, and interpersonal problems were as follows: Ego-resiliency was significantly negatively correlated with aggression (r = −0.21, *p* = 0.005) and interpersonal relationship problems (r = −0.44, *p* < 0.001). Aggression was significantly positively correlated with interpersonal problems (r = 0.30, *p* < 0.001) (Table 4).

### 3.5. Mediating Effects of Aggression on Ego-Resiliency and Interpersonal Problems

The mediating effect of aggression on the effects of ego-resiliency among nursing college students on interpersonal problems is as follows (Table 5). First, as a result of examining the multicollinearity among variables, the Durbin–Watson index was 1.63~1.66, close to 2, indicating that there was residual independence. The VIF (Variance Inflation Factor) index was 1.00 to 1.45, less than 10, making it suitable for regression analysis because there was no problem with multicollinearity.

**Figure 1:**
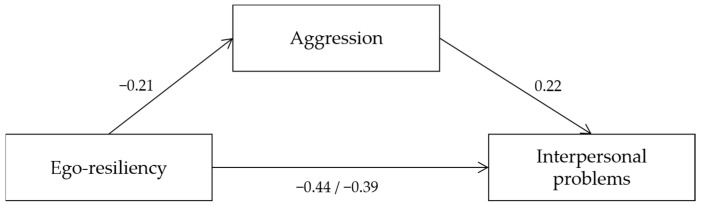
Mediation model of aggression in the relationship between ego-resiliency and interpersonal problems.

To test the mediating effect of ego-resiliency and aggression on interpersonal problems, the three-step verification detailed by Baron and Kenny was conducted. In Step 1, a simple regression analysis of ego-resiliency (independent variable) and aggression (mediating variable) showed statistical significance (β = −0.21, *p* = 0.005). The explanatory power to explain aggression was 4%. In Step 2, the effect on ego-resiliency (independent variable) and interpersonal problems (dependent variable) was found to be statistically significant (β = −0.44, *p* < 0.001). The effect on interpersonal problems was 19%. In Step 3, to test the effect of aggression (mediating variable) on interpersonal problems (dependent variable), a regression analysis was conducted using ego-resiliency and aggression as predictors and interpersonal problems as a dependent variable. As a result, ego-resiliency (β = −0.39, *p* < 0.001) and aggression (β = 0.22, *p* = 0.001) were found to be significant predictors of interpersonal problems. That is, when aggression was used as a mediating variable in Step 3, ego-resiliency was significant in interpersonal problems, and the standardized regression coefficient decreased from −0.44 in Step 2 to −0.39 in Step 3, showing partial mediation (Figure 1).

## 4. Discussion

This study was conducted to investigate the mediating effect of aggression in the effect of ego-resiliency on interpersonal problems in nursing students. Based on the results of this study, we intend to provide basic data for education to strengthen ego-resiliency and reduce aggression to lower the severity of interpersonal problems among nursing students.

In a previous study of second-grade nursing students, their ego-resiliency score was 2.80 points out of 4 [31], but the ego-resiliency of nursing students was 3.38 out of 5 in this study. In this study, a 5-point scale was used to allow for neutrality, and nursing college students were found to have a high level of ego-resiliency, similar to that in the previous study. In addition, in previous studies examining the number of siblings and ego-resiliency, those with more than three siblings showed higher ego-resiliency than did those with two siblings [32]. The ego-resiliency was positively, concurrently correlated with perceived social support from friends and family [33]. In this study, ego-resiliency was higher in the case of two siblings than in the case of one. The number of siblings thus affected ego-resiliency.

In this study, the aggressiveness of nursing college students was low at 1.92 out of 5, and there was no significant difference in relation to general characteristics. In a previous study that analyzed the aggressiveness of general college students using the same tool, male students showed high physical aggression and female students showed anger [26]. Generally, males use more physical aggression and both genders use direct verbal aggression equally [34]. It is thought that nursing college students intend to become nurses, control their aggression, and live in the college area. This can be seen as a difference in aggression from general college students.

In this study, the interpersonal problems score was 2.05 out of 5. The score of first-grade students was higher than those of third- and fourth-grade students, and that of second-grade students was higher than that of fourth-grade students. These results seem to be because, as the students progress through the grades, they meet various people through practice, prepare for national examinations and graduation, and maintain strong relationships with other students, thereby increasing opportunities for interpersonal relationships. In addition, it seems that interpersonal problems decrease as the grade level increases throughout the four years of education. However, in a comparison study, it was found that nursing college students had lower interpersonal scores than did college students with other majors [35]. This is not directly comparable because of the lack of prior studies comparing interpersonal relationship problems between nursing college students and other college students, but in the case of nursing college students, it is considered due to the preparation and practice for nursing national examinations, classes, frequent testing, and job preparation [21]. In comparison, the interpersonal relationship score is considered relatively low, and the interpersonal score is low. In this way, nursing college students’ characteristics linked to the ability to respond flexibly to situational demands such as stress might have consequences regarding emotion regulation and interpersonal problems [36].

As a result of testing the correlations between variables in this study, ego-resiliency was found to be inversely correlated with aggression. This means that ego-resiliency in nursing college students lowers aggression. As such, aggression cannot be controlled, but it can be adjusted by increasing ego-resiliency. Therefore, education and strategies to reduce aggression by identifying and improving an individual’s ego-resiliency are required. In this study, there was a significant inverse correlation between ego-resiliency and interpersonal problems, and there was a significant correlation between aggression and interpersonal problems. These results support the results of a previous study [19,20,33,36]. As such, it was found that the higher the ego-resiliency, the lower the interpersonal problems, and the higher the aggression, the greater the interpersonal problems. Therefore, it was found that aggression has an important effect on interpersonal problems in nursing college students.

Aggression has a partial mediating effect on the relationship between ego-resiliency and interpersonal problems among nursing college students. That is, the higher their ego-resiliency, the lower their aggression; further, the lower their ego-resiliency and the higher their aggression, the greater their interpersonal problems [20]. These variables accounted for 23% of the explanatory power in explaining interpersonal problems, and it was found that ego-resiliency and aggression affect nursing students’ interpersonal problems. As such, it can be seen that the interpersonal relationship problems of nursing college students are a factor influencing aggression in addition to ego-resiliency. Therefore, nursing interventions and education should be provided to reduce aggression [19,20] and create intimate human relationships within mutual relations [33,36].

Nursing students go to college where they live in new student groups and perform a variety of nursing practices. High school students in Korea spend most of their time in school classes and private lessons to prepare for intense college entrance exams. After experiencing the limited interpersonal relationships of high school students, students in the nursing department experience difficulties in interpersonal relationships and express problems. As a result of this study, it was found that increasing the ego-resiliency and decreasing the aggression of nursing students can lower the severity of interpersonal problems [37]. Based on the results of these studies, a program should be developed to help nursing students increase their ego-resiliency and reduce their aggression, which determines their ability to control conflicts in a stressful and threatening environment to maintain positive interpersonal relationships and advance as nurses. Specifically, it is necessary to prepare a positive psychological program that increases ego-resiliency, an anger control program that alleviates aggression, and a mindfulness program [38] that can help students control their minds whenever aggression appears, and to actively mediate the program so that students can easily access it. In addition, through the results of this study, it is possible to grasp the factors of interpersonal problems among nursing college students, so it will be possible to prevent and educate about interpersonal problems by interviewing students. Furthermore, this approach will improve the relationships between nursing college students and help with the complex human relationships formed as a nurse, contributing to the growth of personally mature nurses.

### Limitations

This study was conducted on a modest sample of nursing education students from one city, D City, and this may have limited our ability to detect significant findings. However, the participants were from the nursing college (Baccalaureate) in D province, which was certified by the Korean Accreditation Board of Nursing Education. As such, these findings are reflective of nursing education in the province and appear congruent with global findings. Further investigation with a larger sample is indicated to further validate these findings.

## 5. Conclusions

This study verified aggression as a mediating factor in the relationship between ego-resiliency and interpersonal problems among nursing students. Nursing is the study of providing and practicing care for human beings. This means that the importance and necessity of interpersonal relations among nursing students are greater than those among students of other majors. As a result of the study, it was found that ego-resiliency affects interpersonal problems, aggression plays a part in the relationship between ego-resiliency and interpersonal problems, and the explanatory power is 23%. The results of these studies confirmed that ego-resiliency and aggression are important variables affecting interpersonal problems.

Considering the volume of publications on resilience studies, this study helps to clarify the dimensions of ego-resiliency and shows that the concept still needs to be investigated to further break down the factors of each dimension. Nevertheless, we have found other significant factors in this review, such as ego-resiliency, interpersonal problems, and mediating effects of aggression, which should be considered in future studies and interventions.

Based on the above research results, we would like to suggest further research. First, this study examined limited research subjects in some regions, so it is necessary to repeat the research with an expanded subject group. Second, a research study is suggested to identify other factors affecting interpersonal problems. Third, based on the results of this study, it is suggested to develop and apply a program to improve ego-resiliency and control aggression to reduce interpersonal problems among nursing college students.

## Figures and Tables

**Table 1:** General characteristics of the subjects.

			(*N* = 182)
Variable	Categories	*n*	%
Gender	Female	150	82.4
	Male	32	17.6
Grade	1	29	15.9
	2	45	24.7
	3	48	26.4
	4	60	33.0
Religion	No	112	61.5
	Yes	70	38.5
Number of Siblings	1	19	10.4
	2	121	66.5
	≥3	42	23.1
Education level (father)	≤High school	60	33.0
	Undergraduate	97	53.3
	≥Graduate	25	13.7
Education level (mother)	≤High school	80	44.0
	Undergraduate	93	51.1
	≥Graduate	9	4.9
Economic status	High	56	30.8
	Middle	112	61.5
	Low	14	7.7

**Table 2:** Descriptive statistics among the variables.

Variable	Range	M	SD
Ego-resiliency	1~5		
Ego-resiliency		3.38	0.53
Aggression	1~5	1.92	0.48
Physical Aggression		1.69	0.58
Verbal Aggression		2.16	0.67
Anger		2.27	0.68
Hostility		1.82	0.57
Interpersonal problems	1~5	2.05	0.51
Domineering		1.70	0.62
Vindictive		1.70	0.67
Cold		1.89	0.87
Socially avoidant		2.04	0.81
Nonassertive		2.24	0.85
Exploitable		2.19	0.79
Overly nurturing		2.55	0.73
Intrusive		2.09	0.69

**Table 3:** Differences in ego-resiliency, aggression, and interpersonal problems according to the general characteristics of the participants.

Variable	Categories	N	Ego-Resiliency	Aggression	Interpersonal Problems
			M ± SD	M ± SD	M ± SD
Gender	Female	150	47.20 ± 7.22	50.87 ± 11.11	82.14 ± 20.63
	Male	32	47.47 ± 8.43	50.87 ± 19.20	81.47 ± 18.38
	t/F(*p)*		−0.186 (0.853)	−1.708 (0.096)	0.170 (0.865)
Grade	1 ^a^	29	45.00 ± 7.96	53.55 ± 13.40	94.52 ± 20.67
	2 ^b^	45	49.18 ± 7.23	53.69 ± 13.85	85.87 ± 19.28
	3 ^c^	48	46.29 ± 6.97	52.35 ± 10.62	80.83 ± 17.61
	4 ^d^	60	47.65 ± 7.40	49.48 ± 13.92	74.05 ± 19.27
	t/F(*p)*		2.274 (0.082)	1.146 (0.332)	8.308 (<0.001)a > c, d ^†^, b > d ^†^
Religion	No	112	47.80 ± 7.97	51.59 ± 12.64	82.65 ± 20.76
	Yes	70	46.35 ± 6.40	52.47 ± 13.74	81.01 ± 19.38
	t/F(*p)*		1.282 (0.202)	−0.443 (0.658)	0.531 (0.596)
Number of Siblings	1 ^a^	19	42.84 ± 9.97	55.79 ± 11.17	90.16 ± 25.32
	2 ^b^	121	47.89 ± 7.18	52.17 ± 13.19	80.66 ± 17.74
	≥3	42	47.38 ± 6.18	49.48 ± 13.18	82.26 ± 23.73
	t/F(*p)*		3.937 (0.021)a < b ^†^	1.607 (0.203)	1.835 (0.163)
Education level(father)	≤High school	60	46.75 ± 7.53	52.90 ± 16.29	83.48 ± 18.23
	Undergraduate	97	47.21 ± 7.49	52.52 ± 11.47	83.03 ± 21.78
	≥Graduate	25	48.60 ± 6.99	47.32 ± 8.84	74.60 ± 17.25
	t/F(*p)*		0.549 (0.578)	1.843 (0.161)	1.986 (0.140)
Education level(mother)	≤High school	80	46.31 ± 7.77	53.91 ± 16.12	83.20 ± 18.15
	Undergraduate	93	48.27 ± 7.26	50.22 ± 10.07	81.18 ± 21.87
	≥Graduate	9	45.00 ± 4.06	52.00 ± 6.54	80.22 ± 21.30
	t/F(*p)*		1.949 (0.145)	1.743 (0.178)	0.250 (0.779)
Economic status	High ^a^	56	48.25 ± 6.54	51.48 ± 13.08	79.50 ± 18.10
	Middle	112	47.06 ± 7.41	51.84 ± 12.76	81.68 ± 21.01
	Low ^c^	14	44.71 ± 10.25	54.43 ± 15.69	94.86 ± 18.00
	t/F(*p)*		1.368 (0.257)	0.290 (0.748)	3.363 (0.037)a < c ^†^

^†^ *p* < 0.05 by Scheffé’s post hoc test.

**Table 4:** Correlations among the variables.

Variable	Ego-Resiliency	Aggression	Interpersonal Problems
	r (*p*)	r (*p*)	r (*p*)
Ego-resiliency	1		
Aggression	−0.21 (0.005)	1	
Interpersonal problems	−0.44 (<0.001)	0.30 (<0.001)	1

**Table 5:** Mediating effect of aggression on the relationship between ego-resiliency and interpersonal problems.

Step				B	β	t	*p*	Adj. R^2^	F	*p*
Step 1.	Ego-resiliency	→	Aggression	−0.36	−0.21	−2.84	0.005	0.04	8.09	0.005
Step 2.	Ego-resiliency	→	Interpersonal problems	−1.20	−0.44	−6.56	<0.001	0.19	42.93	<0.001
Step 3.	Ego-resiliency Aggression	→	Interpersonal problems	−1.07	−0.39	−5.90	<0.001	0.23	27.90	<0.001
				0.34	0.22	3.25	0.001			

## Data Availability

The data used is confidential, and the study participants have not consented to data sharing. Due to the sensitive nature of the personal information in the questions asked in this study, the survey respondents were assured that the raw data would be kept confidential and would not be shared.

## References

[1] Marilyn J., Hockenberry D.W. (2018). Wong’s Nursing Care of Infants and Children.

[2] Kwon J.D. (2014). Human Behavior and Social Environment.

[3] Choi S.H. (2018). Effects of ego resilience, interpersonal relation, and cognitive emotion regulation strategies on college life adaptation of nursing students. J. Korea Acad. Industr. Coop. Soc..

[4] Rouse R.A., Al-Maqbali M. (2014). Identifying nurse managers ‘essential communication skills: An analysis of nurses’ perceptions in Oman. J. Nurs. Manag..

[5] Byeon Y.S., Kim M.Y. (2009). Interpersonal conflict experiences of nurses. J. Qual. Res..

[6] Yeun E.J., Kim H. (2013). Development and Testing of a Nurse Turnover Intention Scale (NTIS). J. Korean Acad. Nurs..

[7] Im B.-M., Park J.-M., Kim M.-J., Kim S.-Y., Maeng J.-H., Lee L.-L., Kang K.-A. (2015). A Phenomenological Study on the Turnover Experience of Novice Nurses Working in General Hospital. Korean J. Occup. Health Nurs..

[8] Kim S.M., Suh K. (2015). Relationships between Covert Narcissism and SNS Addiction Proneness: Focus on the Mediating Effects of Experiential Avoidance. Korean J. Health Psychol..

[9] Huh J.E., Chang H.A. (2016). The effect of maternal reaction to children’s negative emotions on interpersonal problems of university students: A mediating effect of internalized shame. Cogn. Behav. Ther. Korea.

[10] Lee Y.O., Joen Y.H., Kim M.S. (2018). Effect of Interpersonal Relation, Ego Resilience and Self-Leadership on Nursing Student’s Adaptation to College Life. J. KSLES.

[11] Lee E.K., Park J.A. (2013). Ego-Resilience and the Clinical Competence of Nursing Students. J. Korean Public Health Nurs..

[12] Shin E.J., Park Y.S. (2013). Emotional Intelligence, Ego Resilience, Stress in Clinical Practice of Nursing Students. J. Korea Acad. Indust. Coop. Soc..

[13] Klohnen E.C. (1996). Conceptual analysis and measurement of the construct of ego-resiliency. J. Pers. Soc. Psychol..

[14] Lee W.S., Shin S.Y. (2014). Structural equation modeling on early childhood teachers’ emotional labor, ego resiliency, job satisfaction. Korean J. Child Educ..

[15] Kim H.S., Kim Y.G., Kim J.U. (2016). The effects of self-differentiation and ego-resilience on interpersonal problem-solving ability of undergraduate students. KALCI.

[16] Han H.R., Lee J.M. (2019). Effect of depressive vulnerability on interpersonal problems among university students: Focusing on moderating effect of ego-resilience. Fam. Environ. Res..

[17] Lee E.A., Cheon S.M. (2013). Development and validation of aggression scale for elementary school students. Korean J. Couns. Psychother..

[18] Perry D.C., Williard J.C., Perry L.C. (1990). Peers’ Perceptions of the Consequences That Victimized Children Provide Aggressors. Child Dev..

[19] Lee W.S. (2018). The Influence of College Students’ Social Withdrawal on Satisfaction with Life: With Emphasis on the Mediating Effect of Aggression. Health Soc. Welf. Rev..

[20] Cho S.H., Ka Y.H. (2020). The relationship between covert narcissism and interpersonal problems of Christian college students focusing on the mediating effect of displaced aggression. Korean Assoc. Christ. Couns. Psychol..

[21] Jun W.-H., Lee G. (2017). The role of ego-resiliency in the relationship between social anxiety and problem solving ability among South Korean nursing students. Nurse Educ. Today.

[22] Block J., Kremen A.M. (1996). IQ and ego-resiliency: Conceptual and empirical connections and separateness. J. Pers. Soc. Psychol..

[23] Yoo S.K., Shim H.W. (2002). Psychological protective factors in resilient adolescents in Korea. Korean J. Edu. Psychol..

[24] Simon H.A. (2012). Models of Discovery: And Other Topics in the Methods of Science.

[25] Buss A.H., Perry M. (1992). The Aggression Questionnaire. J. Pers. Soc. Psychol..

[26] Seo S.G., Kwon S.M. (2002). Validation study of the Korean version of the aggression questionnaire. Kor. J. Clin. Psychol..

[27] Horowitz L.M., Rosenberg S.E., Baer B.A., Ureño G., Villaseñor V.S. (1988). Inventory of interpersonal problems: Psychometric properties and clinical applications. J. Consult. Clin. Psychol..

[28] Alden L.E., Wiggins J.S., Pincus A.L. (1990). Construction of Circumplex Scales for the Inventory of Interpersonal Problems. J. Pers. Assess..

[29] Hong S.H., Park E.Y., Kim Y.H., Kwon J.H., Cho Y.R., Kin Y.K. (2002). Short form of the Korean inventory of interpersonal problems circumplex scales(KIIP-SC). Korean J. Clin. Psychol..

[30] No K.S. (2014). Statistical Analysis: SPSS & AMOS 21.

[31] Park S.-H., Han S.-H. (2016). Effect of Self-resilience and Professional Self-concept, Major satisfaction on Nursing Student’s Adjustment to college life. J. Korea Acad. Coop. Soc..

[32] Ji E.J., Bang M.R., Jeon H.J. (2013). Ego Resilience, Communication Ability and Problem-Solving Ability in Nursing Students. J. Korean Acad. Soc. Nurs. Educ..

[33] Taylor Z.E., Doane L.D., Eisenberg N. (2014). Transitioning from high school to college: Relations of social support, ego-resiliency, and maladjustment during emerging adulthood. Emerg. Adulthood.

[34] Björkqvist K. (2018). Gender differences in aggression. Curr. Opin. Psychol..

[35] Yoon H.S., Kim G.-H., Kim J. (2011). Effectiveness of an Interpersonal Relationship Program on Interpersonal Relationships, Self-esteem, and Depression in Nursing Students. J. Korean Acad. Nurs. Adm. Acronym..

[36] Alessandri G., De Longis E., Eisenberg N., Hobfoll S.E. (2020). A multilevel moderated mediational model of the daily relationships between hassles, exhaustion, ego-resiliency and resulting emotional inertia. J. Res. Pers..

[37] Cho Y.M. (2022). The effects of psychological safety and physical self-concept on ego-resilience in nursing college students. J. Digit. Converg..

[38] Lee M.-R. (2022). The relationship of personality, gratitude, empathy and resilience in nursing students. J. Korean Data Inf. Sci. Soc..

